# Glycosaminoglycan-Based CD44-Targeted Nanoparticles for Cancer Therapy: Biological Barriers and Barrier-Informed Design Strategies

**DOI:** 10.1021/acs.nanolett.6c02400

**Published:** 2026-10-04

**Authors:** Shangyu Chen, Sheida Dabiri, Yichen Xu, Song Li

**Affiliations:** †Center for Pharmacogenetics, ‡Department of Pharmaceutical Sciences, School of Pharmacy, 6614 University of Pittsburgh, Pittsburgh, Pennsylvania 15261, United States; § UPMC Hillman Cancer Institute, University of Pittsburgh, Pittsburgh, Pennsylvania 15261, United States

**Keywords:** Glycosaminoglycan, CD44-Targeted Nanoparticles, Cancer Therapy, Biological Barriers

## Abstract

Nanoparticle (NP)-based drug delivery has been explored to improve the therapeutic index of anticancer agents by enhancing tumor accumulation and reducing systemic toxicity. Among active targeting approaches, cluster of differentiation 44 (CD44) has attracted attention because of its overexpression in tumor cells and tumor-associated endothelial cells and its involvement in tumor progression and therapeutic resistance. Hyaluronic acid (HA) and other glycosaminoglycan (GAG)-based nanocarriers have been developed to exploit CD44-mediated tumor targeting and cellular uptake. However, the *in vivo* performance of CD44-targeted NPs remains variable because of barriers such as systemic clearance, heterogeneous tumor vasculature and stroma, limited receptor accessibility, competition with endogenous ligands, and inefficient intracellular trafficking or payload release. This Letter summarizes advances in CD44-targeted NP delivery, focusing on barriers at systemic, tumor tissue, and cellular levels. We discuss barrier-informed strategies to optimize ligand presentation, improve penetration, promote intracellular payload release, and highlight translational challenges and opportunities for CD44-targeted nanomedicines.

## Graphical abstract

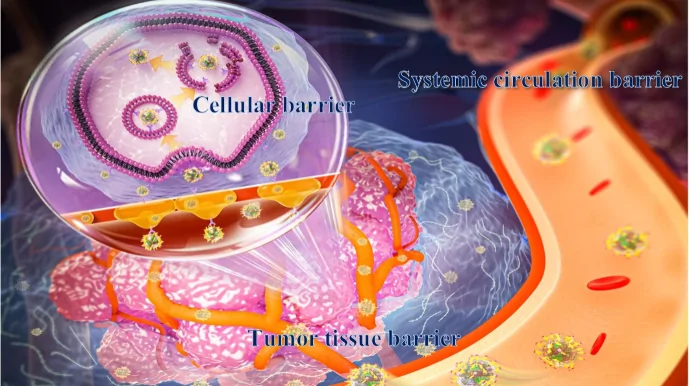

Nanoparticle (NP)-based drug delivery systems have been extensively investigated for cancer detection, prevention, and therapy owing to their potential advantages, including improved drug stability, prolonged blood circulation, enhanced tumor accumulation, and reduced systemic toxicity.^1–5^ Historically, NP accumulation within tumors has been largely attributed to the enhanced permeability and retention (EPR) effect, which results from the leaky vasculature and impaired lymphatic drainage characteristic of tumor tissues.^6–8^ However, despite promising preclinical findings, the EPR effect observed in small-animal xenograft models is often substantially less pronounced in solid tumors of cancer patients.^9^ Furthermore, a recent study by Chan et al. demonstrated that tumor targeting by ligand-free NPs predominantly occurs through an active transendothelial transport mechanism rather than the conventionally recognized EPR-mediated passive accumulation process.^10^

Nevertheless, most reported active targeting strategies rely on NPs functionalized with tumor-specific ligands. Such surface modification is designed not only to improve tumor accumulation but also to facilitate subsequent ligand-mediated intracellular delivery to tumor cells or other target cells upon reaching the tumor microenvironment. Numerous preclinical studies have demonstrated the potential of these active-targeting NPs to enhance tumor targeting and therapeutic efficacy.^11^ Despite these advances, NP delivery occurs within highly complex biological systems and remains hindered by multiple biological barriers at the systemic, tumor, and cellular levels.

Cluster of differentiation 44 (CD44) is a nonkinase transmembrane glycoprotein and one of the most extensively studied receptors for saccharide-based ligands. CD44 is overexpressed in a wide range of tumor cells and tumor endothelial cells (TECs) and is widely recognized as a cancer stem cell marker associated with tumor progression, metastasis, therapeutic resistance, and poor prognosis.^12^ In addition, CD44 plays important roles in regulating endothelial cell functions,^13^ including angiogenesis and vascular remodeling, further supporting its potential as a therapeutic target for NP-based cancer therapy. Several CD44-directed therapeutic strategies have advanced into clinical evaluation, underscoring the translational relevance of this target.^14^

Although several comprehensive reviews have summarized advances in CD44-targeted NPs for cancer therapy, this body of work has largely centered on NP development, targeting performance, and preclinical therapeutic applications.^15–18^ However, the sequential biological barriers encountered during *in vivo* delivery have not been systematically connected with the design strategies needed to overcome them, particularly at the tumor–vascular, tissue, and cellular interfaces. This Letter attempts to address this gap by integrating CD44 biology, glycosaminoglycan (GAG)-based ligand design, NP engineering, and multilevel delivery barriers into a unified barrier-informed framework. Following a brief overview of CD44 biology and hyaluronic acid (HA)-based and other GAG platforms, we examine the barriers affecting systemic circulation, tumor accumulation and penetration, cellular uptake, and intracellular trafficking, together with corresponding design considerations. We conclude by discussing the translational challenges and future directions for the development of more effective CD44-targeted NPs. An overview of this framework is presented in Figure 1.

**Figure 1:**
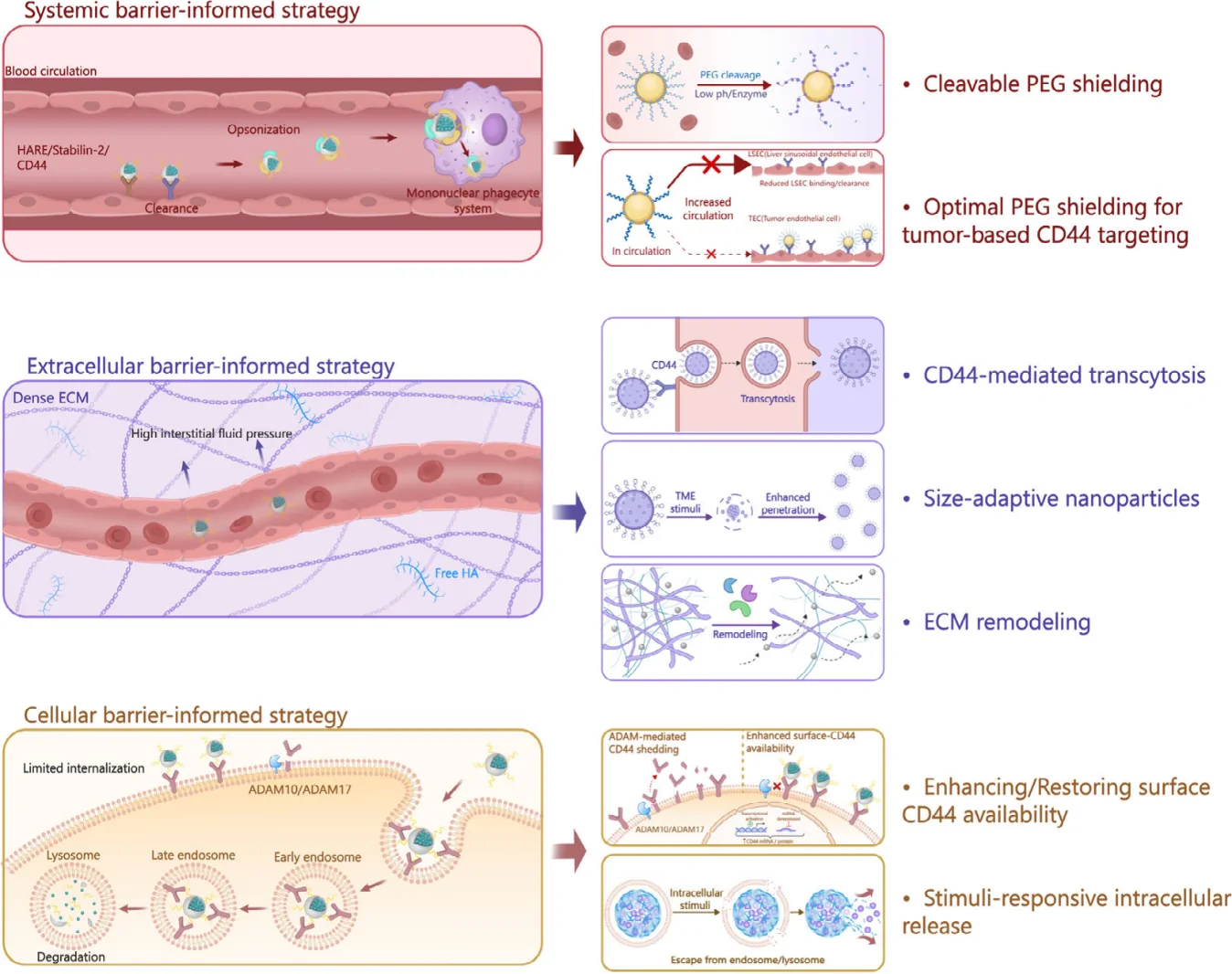
Schematic illustration of the biological barriers and barrier-informed design strategies for GAG-based CD44-targeted NPs delivery.

Understanding the biology of CD44 provides a foundation for examining delivery barriers. CD44 is a transmembrane glycoprotein encoded by a gene on chromosome 11p13 ^19^ and expressed in multiple cell types, including immune cells, fibroblasts, endothelial cells, smooth muscle cells, and epithelial cells.^20^ Structurally, CD44 contains an extracellular ligand-binding domain, a membrane-proximal stem region, a transmembrane domain, and a cytoplasmic tail.^20^ The extracellular domain mediates ligand recognition; the transmembrane domain supports membrane localization and interaction with other proteins, and the cytoplasmic tail links CD44 to intracellular signaling and cytoskeletal adaptor proteins. For NP-based drug delivery, the significance of CD44 lies not only in its elevated expression in tumors but also in its cell-type-dependent ligand accessibility, membrane distribution, and internalization behavior. Within the tumor microenvironment, CD44 is expressed on both tumor cells and TECs, and these two cellular compartments may play distinct roles during NP delivery. CD44 expressed on TECs can mediate the initial interaction of circulating NPs with the tumor vasculature and influence endothelial adhesion or transendothelial transport. In contrast, CD44 expressed on tumor cells primarily contributes to the subsequent binding and intracellular uptake of NPs after they extravasate into tumor tissue. Therefore, both the vascular and tumor cell compartments should be considered in the design of CD44-targeted NP delivery systems.

CD44 exhibits substantial structural heterogeneity because of alternative splicing and post-translational modifications. The diversity of CD44 isoforms is particularly important, as the expression of specific isoforms has been closely associated with disease progression and tumor development.^21–23^ The standard isoform, CD44s, is composed exclusively of constant exons, whereas CD44 variant isoforms (CD44v) contain one or more alternatively spliced variant exons. Although all CD44 isoforms possess an extracellular hyaluronan binding domain, the variant regions can introduce additional interaction sites for growth factors, cytokines, matrix metalloproteinases, and membrane receptors. Consequently, differences in CD44 isoform composition may affect ligand accessibility, receptor clustering, membrane localization, and internalization behavior, all of which are critical determinants of whether CD44 binding can ultimately be translated into efficient NP uptake.

CD44 activity is further regulated by glycosylation, disulfide bond status, epigenetic mechanisms, and proteolytic cleavage. Glycosylation and disulfide-bond formation can modulate ligand-binding affinity, whereas CpG island methylation and microRNAs (miRNAs) may regulate CD44 expression levels.^24,25^ Proteolytic cleavage of the extracellular domain by metalloproteinases generates soluble CD44 (sCD44), which can be detected in biological fluids and has been explored as a diagnostic and prognostic biomarker in several cancers.^26,27^ From the perspective of CD44-targeted nanocarriers, sCD44 may also act as a soluble decoy receptor by competing with membrane-bound CD44 for ligand binding, thereby reducing the effective receptor engagement on target cells. Subsequent cleavage and release of the intracellular domain can further contribute to the intracellular signaling and transcriptional regulation.

In addition, ligand binding can induce conformational changes and promote interactions between the CD44 cytoplasmic tail and adaptor proteins such as ezrin-radixin-moesin (ERM), thereby linking CD44 to cytoskeletal remodeling and intracellular trafficking pathways. Studies have also shown that ADAM17-mediated cleavage of CD44 at the leading edge of migrating cells facilitates cell motility by enabling mechanical stretching and migration through the extracellular matrix.^26^ Collectively, these regulatory mechanisms are highly relevant to the design of CD44-targeted NPs, as they may influence the abundance of accessible receptors on the cell surface, the strength and stability of CD44-ligand interactions, and the intracellular trafficking and fate of CD44-bound nanocarriers.

These receptor-level considerations make ligand selection a critical parameter for CD44-mediated targeting, influencing the biodistribution, tumor penetration, cellular uptake efficiency, safety, and translational potential. As outlined above, successful CD44-targeted delivery depends not only on receptor overexpression but also on ligand accessibility, binding dynamics, internalization pathways, and receptor turnover.^28^ In NP-based platforms, CD44 targeting can simultaneously interfere with tumor progression-related signaling and enhance therapeutic cargo delivery. Thus far, most studies have primarily correlated therapeutic efficacy with targeting efficiency, while the potential effects of NP-CD44 engagement on CD44-mediated signaling and its contribution to overall therapeutic outcomes have received comparatively less attention.

A variety of CD44-targeting ligands have been developed, including peptides, antibodies, aptamers, and GAG-based ligands. Several representative CD44-targeted therapeutic strategies have entered clinical evaluation, ranging from early phase studies to Phase III trials (Table 1). CD44-binding peptides such as A6/SPL-108 have entered clinical studies as antimetastatic agents;^29^ however, peptide-functionalized CD44-targeting nanocarriers remain largely at the preclinical stage. Similarly, CD44v6-targeted antibody–drug conjugates, such as bivatuzumab mertansine, demonstrated tumor-targeting capability and partial clinical responses, but their development was ultimately limited by severe on-target/off-tumor toxicity due to CD44v6 expression in normal tissues.^30^ In addition, several HA-based drug conjugates have also progressed into clinical evaluation, suggesting the translational potential of GAG-based delivery systems (Table 1).^31–33^ Collectively, these clinical experiences highlight both the promise and limitations of CD44-targeted strategies and have driven increasing interest toward rationally designed GAG-based nanocarriers, which are the focus of the remainder of this Letter.

**Table 1:** Representative CD44-Targeted Therapeutic Strategies Evaluated in Clinical Studies

Category	Strategy	Cancer type	Phase	Major finding	Trial ID
CD44-binding peptide	A6/SPL-108	Ovarian cancer and other metastatic solid tumors	Clinical evaluation	Entered clinical studies as an antimetastatic agent; however, peptide-functionalized CD44-targeting nanocarriers remain largely preclinical	NA
Anti-CD44 antibody	Bivatuzumab mertansine	CD44v6-positive HNSCC, breast cancer, esophageal SCC	Phase I	Demonstrated tumor-targeting capability and partial clinical responses, but development was discontinued due to severe on-target/off-tumor toxicity	NCT02254018
					NCT02254005
					NCT02254031
					NCT02254044
	RG-7356	AML or advanced solid tumors	Phase I	Early clinical evaluation; limited clinical validation for nanocarrier-based delivery	NCT01641250
					NCT01358903
HA-drug conjugate	FOLF(HA)-Iri	Metastatic colorectal cancer	Phase III	Improved PFS, but no significant improvement in OS or toxicity profile	NCT01290783
	CA102N	Advanced or metastatic solid tumors; metastatic colorectal cancer	Phase I	Safe and tolerated at RP2D; antitumor efficacy remains exploratory	NCT03616574
	ONCOFID-P-B	BCG-unresponsive bladder carcinoma in situ, with or without Ta/T1 disease	Phase III	Local administration; prior Phase I/II study reported complete responses in a subset of patients	NCT05024773

Of the CAG-based ligands, HA, the natural ligand of CD44, has been the most extensively investigated for targeted delivery applications.^24,25,34^ HA is particularly attractive because of its biocompatibility, biodegradability, chemical versatility, scalability, and dual functionality as both a targeting ligand and a structural component of drug carriers. For instance, its polyanionic nature enables electrostatic complexation with cationic hydrophobic macromolecules, allowing self-assembly into NP systems for drug delivery.^15^ In addition, HA can be conjugated with hydrophobic chemotherapeutics such as doxorubicin (DOX), paclitaxel (PTX), docetaxel (DTX), and camptothecin (CPT) to form prodrugs with improved tumor accumulation and penetration, followed by CD44-mediated uptake and subsequent intracellular release of active therapeutic moieties inside tumor cells. Notably, HA-drug conjugates, including FOLF­(HA)-Iri, CA102N, and ONCOFID-P-B, have entered clinical evaluation with some demonstrating acceptable safety profiles and progression into later-stage trials (Table 1),^31–33^ supporting HA-based systems as a feasible translational route for CD44-targeted drug delivery.

However, HA-based targeting is biologically complex, as HA is not an inert ligand but an endogenous polysaccharide capable of multivalent interactions with multiple HA-recognizing receptors and extracellular matrix components. In addition to CD44 binding, HA can interact with other HA-binding receptors and matrix constituents, thereby influencing circulation behavior, biodistribution, tumor penetration, and cellular uptake.^35,36^ Consequently, the performance of HA-based nanocarriers is determined not only by CD44 affinity but also by HA molecular weight, surface density, spatial presentation, and the *in vivo* biological context.

Chondroitin sulfate (CS) represents another important non-HA GAG platform for CD44-related delivery. CS shares structural similarity with HA and has been reported to undergo CD44-mediated endocytosis, supporting its use as a CD44-interacting ligand for nanocarrier design.^14^ Compared with nonsulfated HA, CS contains sulfate groups that confer additional anionic character, sulfation-dependent binding properties, and distinct interactions with extracellular matrix components. These features may favor electrostatic complexation with cationic polymers or polyplex-forming materials, enabling the self-assembly of CS-based NPs or polyplexes for drug and gene delivery. Consistently, CS-modified PEI systems have been shown to reduce PEI-associated cytotoxicity while maintaining efficient gene transfection through CD44-mediated uptake.^37^ From a biological perspective, CS may also regulate the CD44-associated adhesion and invasion signaling. This is because CD44 itself can carry CS modifications, forming a CS-modified proteoglycan that contributes to tumor cell invasiveness through integrin-related pathways.^38^ Therefore, CS can be viewed as a distinct but complementary GAG platform alongside HA for CD44 targeting.

In addition to HA and CS, other sulfated GAGs, such as heparan sulfate (HS), may influence the CD44-related biology. Although HS has been explored as a biomaterial scaffold for drug delivery, these applications do not rely on direct CD44-targeting. Its relevance lies mainly in CD44 variant biology, particularly the HS modification of CD44v3 ^39–41^ and its role in presenting heparin-binding growth factors.^42^

Collectively, these findings suggest that effective GAG-based CD44 targeting depends on more than ligand–receptor affinity and should consider the sequential biological barriers that influence NP delivery, providing a rationale for a barrier-informed design approach.

Following systemic administration, GAG-based NPs must navigate multiple barriers, including systemic clearance, tumor accumulation and penetration, receptor accessibility, cellular uptake, and intracellular trafficking and release (Table 2). Accordingly, GAG ligand properties and NP physicochemical characteristics can be considered together to address these barriers at different stages of the delivery process.

**Table 2:** Barrier-Informed Design Strategies for GAG-Based CD44-Targeted NPs

Biological barrier	Key delivery challenge	Barrier-informed design strategies	Ref
Systemic circulation level	Premature clearance; hepatic scavenger receptor- and HA receptor-mediated on-target, off-tumor uptake	Tune HA/GAG molecular weight and density; use PEG/PEG-GAG or albumin-based shielding; introduce stimuli-responsive deshielding to preserve circulation while restoring CD44 recognition at tumor site	^43–48^
Tumor tissue level	Limited extravasation, dense ECM, high interstitial pressure, and restricted CD44 accessibility	Exploit CD44-mediated transcytosis; optimize size/charge; use HAase-, pH-, or enzyme-responsive size switching, ECM remodeling, or strategies that enhance intratumoral retention	^49–52^
Cellular level	Inefficient productive internalization, endosomal trapping, lysosomal degradation, and limited intracellular payload release	Optimize CD44 density and surface accessibility; incorporate endosomal escape modules and pH/redox/ROS/enzyme-responsive release systems to convert CD44 binding into therapeutic function	^53–57^

The first of these barriers is rapid blood clearance following systemic administration regardless of whether NPs carry targeting ligands. For most NP systems, this is initiated by opsonin adsorption followed by recognition by the mononuclear phagocyte system (MPS).^58^ This process can be reduced by surface shielding with hydrophilic polymers such as polyethylene glycol (PEG), which suppresses protein adsorption and prolongs circulation time.^58^ HA-modified NPs can also decrease opsonization because of their highly hydrated and sterically protective surface. However, unlike inert stealth coatings, HA-based systems introduce additional biologically active clearance pathways. This is particularly relevant because circulating HA is physiologically cleared via hepatic and lymphatic routes.^34^ In this context, HA-decorated NPs may be recognized by HA-binding receptors in nontumor tissues, including HARE/stabilin-2 and LYVE-1, as well as CD44 on liver-associated and immune cells. HARE/stabilin-2, highly expressed on liver sinusoidal endothelial cells (LSECs), serves as a major scavenger receptor for circulating HA.^28,59^ Consequently, the disposition of HA-based NPs may be influenced by both nonspecific MPS uptake and HA-receptor-mediated clearance in nontumor tissues.

PEG modification can reduce these interactions and prolong circulation by limiting both opsonin adsorption and HA-receptor recognition;^60^ however, it may also compromise targeting to CD44-expressing tumor cells and TECs. Notably, although HA can interact with both TECs and LSECs, the CD44 expression is generally higher in tumor-associated cells, making them less susceptible to PEG-mediated inhibition. Therefore, optimizing the degree of PEG shielding may preferentially inhibit HA binding to LSECs, thereby reducing liver clearance and increasing the availability of NPs in the tumor circulation for a subsequent interaction with TECs.

This trade-off highlights the need for surface engineering strategies that decouple systemic stability from receptor recognition at the tumor site. Optimized or dynamic shielding can suppress premature clearance in circulation while preserving CD44 accessibility in CD44-rich tumor regions, thereby enhancing selective uptake by CD44-high cells over CD44-low cells. Consistent with this concept, tuning PEG density in HA- or CS-containing NPs has been shown to reduce uptake by Kupffer cells and LSECs while preserving tumor cell interactions, indicating partial decoupling of clearance and targeting. Similarly, in our PMBOP-CP nanocarrier system,^61^ PEG-CS optimization reduced LSEC uptake more strongly than uptake by the CD44-high TEC counterpart, increasing circulation time while maintaining tumor endothelial targeting.

However, excessive shielding may also interfere with receptor recognition and cellular uptake. Stimuli-responsive shielding provides a different solution by temporally separating the circulation phase from the tumor-recognition phase. For example, a Dox-loaded transformable HA-based supramolecular nanoplatform used an acid-labile benzoic imine linkage to attach a detachable PEG shell.^44^ At physiological pH, the PEG layer buried HA ligands and reduced nonspecific RES capture, allowing the system to remain in a “stealth” state during circulation. In the acidic tumor microenvironment, PEG detachment exposed HA and converted the particles into a “recognition” state, thereby restoring CD44-mediated tumor-cell uptake. This strategy highlights how dynamic surface transformation can balance PEG-mediated systemic stability with HA-mediated receptor recognition at the tumor site.

HA molecular weight and surface presentation are additional determinants of systemic behavior.^34^ Longer HA chains or densely grafted HA can provide multivalent binding sites that promote stronger CD44 interaction and tumor cell internalization, but these same features may also increase recognition by HA clearance pathways and shorten circulation. Conversely, shorter HA chains or lower grafting density may reduce clearance but weaken the CD44-binding avidity. This trade-off was demonstrated in HA-grafted liposomes with different HA polymer lengths, where HA modification enhanced CD44-dependent uptake but compromised blood circulation and tumor accumulation compared with PEGylated liposomes.^43^ Importantly, the dual PEG/HA coating preserved PEG-like circulation and tumor accumulation while improving internalization by CD44-positive tumor cells. Together, these findings highlight the need to balance HA-mediated CD44 recognition with prolonged systemic circulation rather than maximizing the ligand density or binding avidity alone.

Even when circulation and tumor accumulation are improved, CD44-targeted NPs must still overcome tissue-level barriers to reach target cells beyond the tumor vasculature. As discussed above, tumor vasculature is not only a physical barrier to NP extravasation but also an active biological interface that regulates NP–tumor interactions. For GAG-based CD44-targeted NPs, the overall tumor targeting may therefore result from both EPR-assisted accumulation and an active interaction with CD44-expressing TECs. However, vascular-level accumulation alone does not guarantee an effective intratumoral delivery. The key challenge at the tumor tissue level is to convert tumor accumulation into deeper tissue penetration and functional access to CD44-positive target cells.^62^

One strategy for addressing this barrier is to exploit CD44-mediated transendothelial transport. Our group previously developed biodegradable chitosan NPs coated with CS/PEG-CS for the codelivery of presiRNA and DOX.^47^ Using WT and CD44-knockout (KO) mice bearing WT or CD44-KO tumors, together with transwell and tumor spheroid models, this study investigated the contribution of CD44-mediated transport to tumor targeting and penetration. Whole-body CD44 KO markedly reduced NP accumulation in both WT and CD44-deficient tumors with a more pronounced reduction in CD44-deficient tumors. Moreover, in WT mice bearing CD44-deficient tumors, NPs were largely restricted to perivascular regions and showed a limited tissue penetration. These findings indicate that CD44 contributes not only to tumor cell uptake but also to endothelial transport and intratumoral penetration.

This process may be conceptualized as a two-step CD44-associated delivery model (Figure 2). In the first step, GAG-coated NPs engage CD44-expressing TECs, facilitating endothelial binding and internalization and potentially contributing to extravasation across the tumor vascular barrier via transcytosis. In the second step, following extravasation, CD44 recognition on tumor cells may promote receptor-mediated uptake, transcellular transport, and intratumoral redistribution, contributing to the tumor penetration. This second step is supported by our findings that GAG-coated NPs, but not uncoated NPs, penetrated tumor spheroids and reached the spheroid cores. Further support comes from the study of El-Dakdouki et al., which demonstrated that internalized HA-coated silica NPs could be released from tumor cells and subsequently taken up by neighboring cells in multilayered tumor models, leading to enhanced penetration.^63^ Together, these findings suggest that GAG-based CD44 targeting may operate at multiple tumor interfaces, including TEC-mediated vascular interaction, tumor cell uptake, and context-dependent intratumoral redistribution.

**Figure 2:**
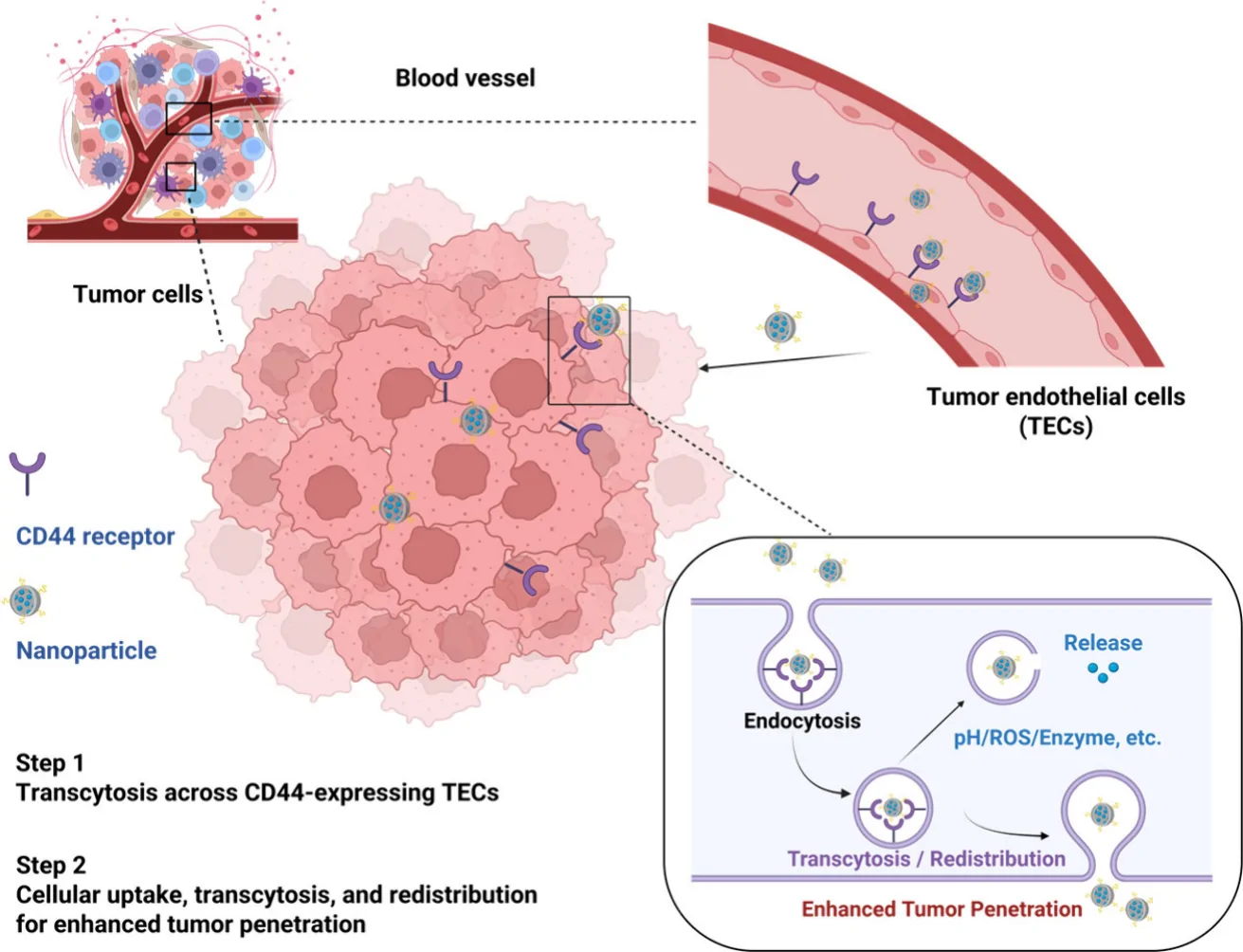
Potential contribution of CD44-mediated transcytosis to multistep tumor penetration.

Nevertheless, CD44 accessibility within tumors is highly context dependent. Many solid tumors contain dense ECM enriched in hyaluronan and cross-linked collagen, which increases interstitial fluid pressure, restricts NP diffusion, and may interfere with ligand–receptor contact. In addition, CD44 undergoes dynamic turnover and proteolytic shedding, generating soluble CD44 that can compete with membrane-bound CD44 for HA- or GAG-coated NPs.^27,64^ Tumor-derived HA or GAG oligosaccharides may further promote CD44 cleavage through protease-dependent mechanisms, suggesting that the tumor matrix can regulate CD44 availability as well as physically restrict NP penetration.^65^ These features create a tumor-tissue-level barrier in which CD44 is present but not always functionally accessible.

Several complementary strategies have been developed to improve the tumor penetration and CD44 accessibility. Size-adaptive HA/HSA hybrid NPs use HAase-triggered HA degradation to convert larger circulating NPs into smaller tumor-penetrating fragments while preserving HA-mediated CD44 targeting.^49^ HA/PEG-cross-linked hierarchical NPs further enable pH-triggered release of hyaluronidase to locally degrade HA-rich ECM under acidic tumor conditions, while PEG cross-linking maintains stability during circulation.^52^ Beyond enzyme-responsive systems, HA-modified nanobots combine active propulsion, HA-mediated CD44 anchoring, and local barrier loosening to overcome diffusion-limited penetration.^50^ Strategies have also been developed to address the unique ECMs of certain tumors. For example, in bone tumors, chondroitin sulfate A-based nanotherapeutics can be combined with alendronate to improve hydroxyapatite-mediated retention while maintaining CD44-mediated tumor-cell uptake.^51^ Collectively, these examples illustrate how GAG-mediated receptor targeting may be complemented by size adaptation, ECM remodeling, nanobot-assisted propulsion, or microenvironment-specific retention to improve tumor level delivery.

Even when GAG-based CD44-targeted NPs penetrate tumor tissue and reach target cells, receptor binding and cellular uptake do not necessarily ensure a functional intracellular delivery. CD44-mediated internalization is often considered to be distinct from classical clathrin- or caveolae-dependent uptake pathways. Several studies have suggested that HA uptake through CD44 can occur largely independently of conventional clathrin- or caveolae-mediated endocytosis, which may be related to the absence of a canonical AP-2 binding sequence in the CD44 cytoplasmic tail.^34^ Instead, CD44 trafficking is closely linked to receptor clustering, lipid-raft localization, and post-translational regulation. The transmembrane domain of CD44 and palmitoylation-dependent localization within cholesterol-rich membrane domains can regulate receptor mobility, ligand interaction, and internalization.^28^ Importantly, disruption of these processes can impair internalization without abolishing ligand binding, indicating that CD44 engagement alone is insufficient to predict productive uptake.

The intracellular route of CD44-targeted nanocarriers may also vary depending on the ligand type, receptor processing, and carrier design. Although HA/CD44 uptake is frequently described as nonclassical, some CD44-targeted systems can involve clathrin-associated endocytosis. For example, camptothecin-loaded CS-*g*-polymer micelles were reported to enter lung cancer cells through a mechanism involving both CD44 and clathrin-mediated endocytosis.^25^ Moreover, we recently reported that knockout or knockdown of iRhom1 or iRhom2 reduced CD44 cleavage and enhanced CD44-mediated tumor targeting of NPs, likely by decreasing ADAM17 activity.^47^ These observations suggest that CD44-targeted NP trafficking is context dependent and may be influenced by ligand presentation, particle structure, receptor clustering, and CD44 cleavage or turnover. Therefore, rational design should consider not only whether NPs bind CD44 but also which intracellular route they enter and whether that route favors payload activity.

After internalization, endosomal trapping, lysosomal degradation, and inefficient cytosolic release become major barriers, particularly for nucleic-acid therapeutics. Incorporating endosomal escape or intracellular release mechanisms is therefore essential for converting CD44-mediated uptake to therapeutic function. For example, the synthetic anionic peptide GALA has been incorporated into HA-based nanogels to promote endosomal membrane disruption and intracellular cargo release.^66^ In another study, CD44-driven uptake of HA-conjugated liposomes in glioblastoma cells was associated with reduced lysosomal colocalization compared with particles internalized by CD44-low or CD44-negative cells, suggesting that CD44 engagement may influence not only uptake efficiency but also intracellular fate.^67^ However, whether CD44 targeting consistently promotes lysosomal evasion remains context dependent and requires further investigation.

Several recent CD44-targeted designs have addressed these intracellular barriers through sequential or stimulus-responsive mechanisms. HA-coated ROS-responsive micelles, for example, used HA for CD44-mediated uptake and lysosomal HAase-triggered removal of the HA shell to expose mitochondrial-targeting TPP groups, followed by ROS-triggered drug release in mitochondria.^53^ HA-SS-PTX/Mn prodrug micelles coupled CD44-mediated uptake with GSH-responsive PTX release and Mn^2+^-mediated STING activation, linking intracellular drug delivery to antitumor immunity.^54^ In another strategy, HA-coated mesoporous silica NPs used CD44-mediated uptake as the entry step, while NIR irradiation triggered ROS/heat/NO generation and Ca^2+^ overload for controlled intracellular activation.^55^ Together, these examples suggest that CD44-targeted NPs should be designed not only to enter cells but also to direct intracellular trafficking, release payloads in response to tumor-specific cues, and activate defined therapeutic pathways.

Despite this preclinical progress, whether these barrier-informed strategies translate into consistent clinical benefit remains uncertain. A major challenge is the gap between promising animal studies and the more variable efficacy observed in patients, which reflects not only the limited and heterogeneous EPR effect in human tumors but also the inability of many systems to overcome multiscale biological barriers.^20^ Clinical experience illustrates both the promise and limitations of this strategy. For example, the CD44v6-targeted antibody–drug conjugate bivatuzumab mertansine showed tumor-targeting potential but was discontinued because of severe toxicity, highlighting the risk of on-target/off-tumor effects.^29,30^ In contrast, HA-based drug conjugates may offer a more biocompatible route: FOLF­(HA)-Iri reached phase III evaluation in metastatic colorectal cancer and improved progression-free survival, although without significant overall survival or toxicity benefit, while CA102N and ONCOFID-P-B entered clinical testing with acceptable early tolerability but still require stronger evidence of broad therapeutic benefit.^31–33^

These outcomes indicate that CD44 overexpression alone is insufficient to ensure the clinical efficacy or safety. As discussed in this Letter, CD44 is a dynamic receptor regulated by isoform diversity, glycosylation, shedding, recycling, and cell-type-specific accessibility, while HA and other GAG ligands are bioactive polymers that influence not only receptor binding but also systemic clearance, biodistribution, tumor penetration, immune interactions, and intracellular trafficking.^14,24,28,44^ Therefore, the performance of CD44-targeted NPs depends on the coordinated interaction among receptor biology, ligand presentation, nanocarrier architecture, and the tumor microenvironment. At the systemic level, premature clearance by hepatic and MPS pathways should be minimized; at the tumor tissue level, vascular transport, ECM penetration, and functional CD44 accessibility need to be improved, and at the cellular level, CD44 binding must be converted to productive intracellular delivery and payload release.

Looking forward, CD44-targeted nanomedicine should benefit from expanding beyond a ligand-centric paradigm to a more barrier-informed engineering approach. Future systems should not simply maximize CD44-binding ligand density but instead balance systemic stability, tumor endothelial interaction, intratumoral penetration, receptor accessibility, intracellular release, immune compatibility, safety, manufacturability, and reproducibility. Because CD44/HA-mediated interactions are not tumor-exclusive, the disease context should be carefully considered. In patients with inflammatory comorbidities such as atherosclerosis, HA-based NPs may accumulate in macrophage-rich plaques, creating competing nontumor reservoirs, although such tropism may also offer therapeutic opportunities for inflammatory diseases.^68^ Therefore, disease-dependent biodistribution, immune interactions, and long-term safety require a careful evaluation. More clinically relevant models, including patient-derived xenografts, humanized mouse models, organoids, and advanced spatial or single-cell analytical tools, may help identify the tumor contexts and patient populations that are most likely to benefit from CD44-targeted platforms. By integration of CD44 biology with GAG ligand engineering and multiscale delivery design, future CD44-targeted nanomedicines may achieve more predictable tumor delivery and clinically meaningful therapeutic outcomes.
